# Public health and research ethics education: the experience of developing a new cadre of bioethicists at a Ugandan institution

**DOI:** 10.1186/s12909-023-04974-y

**Published:** 2024-01-03

**Authors:** Gertrude N. Kiwanuka, Francis Bajunirwe, Paul E. Alele, Joseph Oloro, Arnold Mindra, Patricia Marshall, Sana Loue

**Affiliations:** 1 Faculty of Medicine, Mbarara University of Science and Technology, Mbarara, Uganda; 2 School of Medicine, Case Western Reserve University, Cleveland, OH USA

**Keywords:** Research ethics, Public health, Curriculum, Bioethics education, Training programs, Africa

## Abstract

Research ethics education is critical to developing a culture of responsible conduct of research. Many countries in sub-Saharan Africa (SSA) have a high burden of infectious diseases like HIV and malaria; some, like Uganda, have recurring outbreaks. Coupled with the increase in non-communicable diseases, researchers have access to large populations to test new medications and vaccines. The need to develop multi-level capacity in research ethics in Uganda is still huge, being compounded by the high burden of disease and challenging public health issues. Only a few institutions in the SSA offer graduate training in research ethics, implying that the proposed ideal of each high-volume research ethics committee having at least one member with in-depth training in ethics is far from reality. Finding best practices for comparable situations and training requirements is challenging because there is currently no “gold standard” for teaching research ethics and little published information on curriculum and implementation strategies. The purpose of this paper is to describe a model of research ethics (RE) education as a track in an existing 2-year Master of Public Health (MPH) to provide training for developing specific applied learning skills to address contemporary and emerging needs for biomedical and public health research in a highly disease-burdened country. We describe our five-year experience in successful implementation of the MPH-RE program by the Mbarara University Research Ethics Education Program at Mbarara University of Science and Technology in southwestern Uganda. We used curriculum materials, applications to the program, post-training and external evaluations, and annual reports for this work. This model can be adapted and used elsewhere in developing countries with similar contexts. Establishing an interface between public health and research ethics requires integration of the two early in the delivery of the MPH-RE program to prevent a disconnect in knowledge between research methods provided by the MPH component of the MPH-RE program and for research in ethics that MPH-RE students are expected to perform for their dissertation. Promoting bioethics education, which is multi-disciplinary, in institutions where it is still “foreign” is challenging and necessitates supportive leadership at all institutional levels.

## Introduction

Education on conducting ethical research seeks to increase the number and quality of bioethicists to undertake ethically sound and innovative investigations, equip them with skills for training and mentoring a new generation of scientists and bioethicists, support regulatory structures, and contribute to research policy [1]. In the context of Africa, bioethics education is an emerging field; it is not yet as institutionalized in mainstream academia as it is in Western universities [2]. Consequently, there is less recognition by African institutions and governments, as such, it is mainly funded by international agencies. While the establishment of bioethics education in the West is solid, with higher degree programs and centers making it an academic and professional discipline, and as such, providing insight into international research ethics curricula, we know little about curricula being implemented in SSA, especially in resource-limited settings where the burden of disease is high, providing an environment for research.

Bioethics training programs for Africa started as, and have generally been, internationalized undertakings. The controversial studies on human immunodeficiency virus (HIV)-vertical transmission in low- and middle-income countries (LMIC), which could not be implemented in the West [3, 4], revealed an urgent need to develop research ethics in developing countries to international standards [5]. The US National Institutes of Health Fogarty International Centre (NIH FIC) responded by providing grants to develop international research ethics curricula for in-depth training for professionals and academicians from LMICs [1]. The first institutions to receive these grants were Johns Hopkins University (JHU) in Baltimore, Maryland, USA; Case Western Reserve University (CWRU) in Cleveland, Ohio, USA; the University of Toronto in Canada; and three South African universities – the University of Cape Town and an initial collaboration between the Universities of Pretoria and KwaZulu Natal in association with JHU [6, 7]. Uganda was a beneficiary of all of them, possibly because of the HIV-transmission prevention clinical trials which were considered unethical at the time [8, 9].

Oftentimes, innovative ideas and implementation plans for research ethics training programs flow from experts in high-income countries (HIC) [10] to LMIC. While this allows trainees to acquire skills to function as globally-informed citizens of international standards, there are challenges that have been reported on employability for bioethics graduates [11], limited capacity development in governance [12], the contextualization of research ethics to local needs, and sustainability over the mid-term to long-term [6, 13].

### Historical shaping of bioethics education for Africa

Formal instruction in bioethics focusing on responsible conduct of research (RCR) with a component of scientific integrity commenced in 1989 following a US policy release that mandated the inclusion in every institutional training grant application competing for federal funds a description of formal training in RCR [14]. In line with the Fogarty International Center’s (FIC) strategic plan to strengthen research bioethics expertise in LMIC, the International Bioethics Education and Career Development Award program was released in March 2000. Between 1993 and 2013, UNESCO provided support for the development of national bioethics committees training materials to develop the capacity of their members [15]. The Wellcome Trust of the United Kingdom has also been instrumental in funding training in ethics of biomedical research in developing countries [16], while the European and Developing Countries Clinical Trial Partnership has supported capacity development in research ethics governance, ethical review of clinical research, policy development, and use of medicinal products and technologies in humans since 2015 [17]. The Council on Health Research for Development (COHRED) mapped and provided capacity development for many research ethics committees (RECs) in different countries [18].

### The first five bioethics education programs training Africans

From 2000 to 2017, the Fogarty African Bioethics Training Program (FABTP) at Johns Hopkins University in the USA focused on individuals and offered non-degree training opportunities to researchers, members of RECs, and professionals involved in research from Africa [19]. In 2010, the FABTP shifted its focus to developing institutional research ethics capacity through selected institution partnerships [13]. By 2012, 30 trainees from 14 African countries had completed the JH FABTP [13]. The FABTP evolved into the African Bioethics Consortium, a network of US institutions and three others in Africa, namely: Makerere University, College of Health Sciences in Uganda; the University of Botswana, Office of Research and Development; and the University of Zambia, School of Medicine [20].

The training of African bioethics scholars at CWRU started in 2001, but there was already an ongoing Uganda-CWRU Research Collaboration, established in 1988 with funding from the US NIH, to build capacity and provide training through HIV/AIDS and tuberculosis research to improve care. Between 2001 and 2007, 11 African scholars, mostly physicians (six from Uganda, affiliated with Makerere University, and five from Nigeria), were trained at CWRU (M. Norris, personal communication, March 30, 2023) under the International Research Ethics Training Program (IRETP). The program also supported the development of faculty from collaborating countries to teach research ethics. Faculty members traveled to CWRU to be exposed to teaching contemporary issues in research ethics, mentoring, and to discuss program implementation. In 2008, the program stopped receiving trainees from Africa, given the establishment of bioethics training programs on the continent.

The University of Toronto Masters of Health Sciences in Bioethics (MHSc), International Stream, UTMBIS (2000–2012) focused on capacity-building efforts in international research ethics. African trainees were mainly from Ghana and Nigeria. By 2008, the program had graduated 32 fellows with MHSc degrees [21], including those from India, Pakistan, and Bangladesh.

The International Research Ethics Networks for Southern Africa (IRENSA) at the University of Cape Town, South Africa, offered postgraduate diploma training in international research ethics. By 2011, the program had trained 97 mid-career professionals from South Africa and eight other low-income African countries [6]. Between 2012 and 2015, the program admitted 43 trainees under the Advancing Research Ethics Training in Southern Africa (ARESA) program at Stellenbosch University [22].

The Universities of Pretoria and KwaZulu-Natal were the first African institutions to collaboratively offer a Master’s degree in Health Research Ethics under the South African Ethics Training Initiative (SARETI) [6]. Between 2000 and 2017, the SARETI provided long-term training to trainees from 17 African countries [6] leading to the award of certificates and three Master’s degrees with a track in research ethics (Masters in Public Health, MPH; Master in Philosophy, MPhil; and Masters in Social Sciences, MSocSc). Although the focus of SARETI was on studies at the Masters level, by 2012 the program had graduated one PhD [7]. The MPH program was discontinued in 2013, but more students were registered for the MSocSc. Over 17 years, SARETI produced 22 fellows with certificates: 14 MPH, 2 MPhil, and 36 MSocSc (C. Pettit, personal communication, April 18, 2023), making it a very productive research ethics education program on the African continent.

Former trainees of these bioethics education programs have been instrumental in shaping research ethics in their home countries with notable individual and collective success. Some programs have reported their former trainees supporting the development of national policies and research regulation guidelines, winning bioethics-related grants, and establishing institutional review boards [19], leading and serving on RECs, authoring and coauthoring publications on research ethics [23], developing of curriculum of new programs and teaching [19]. However, there is still a critical need for expertise to teach research ethics [6]. Besides, some former trainees found it difficult integrating their newly acquired knowledge in their home institutions [24]. This is suggestive of the importance of beneficiary institutions in LMIC assuming a rightful place and space to determine research ethics training strategies that address local needs for sustainable development.

Internationalization of bioethics or research ethics programs is premised on the need to integrate an international, intercultural, and global dimension in the training of future bioethicists. The advantage of this is raising awareness of global issues, and helping students develop global perspectives and attitudes. The pitfall is over dependence on faculty and mentors from HIC and developing strategies that may not be contextualized to local examples and needs [11, 25].

### Theoretical background and purpose

The volume of international research conducted in SSA by investigators from HIC is high. Many countries in SSA, including Uganda, have a high burden of infectious diseases like HIV and malaria, providing researchers with access to large sample size for drug trials and behavioral studies [26]. Africa continues to face emerging global health problems, like outbreaks of Ebola and Marburg viruses [27], which require testing new medications and vaccines, but many ethical issues related to such studies remain unresolved [28]. In Uganda, the western and southwestern regions have experienced outbreaks of yellow fever [29], rift valley fever [30], measles [31], and the Ebola virus disease from border crossing with the neighboring Democratic Republic of Congo [32], creating a perfect environment that requires responses from public health experts and researchers. Furthermore, Uganda is facing an epidemiological transition in disease burden from communicable to non-communicable diseases [33]. While there are ethical dilemmas that health professionals face in decision-making during practice, necessitating training in bioethics in clinical, biomedical, and health sciences, the need to develop capacity in research ethics in Uganda is still huge, being compounded by the high burden of disease and challenging public health issues. A study in Uganda revealed disturbing proportions of REC members with low competence to review studies on controlled human infection models, reverse pharmacology design, and new technology and digital health interventions [34]. With only a few institutions in Africa offering higher-degree training in research ethics, the proposed ideal of each high-volume REC having at least one member with in-depth training in ethics [35] is far from reality. Despite the fact that some training programs are more popular than others and have significant in-country expertise, there is no “gold standard” curriculum for teaching research ethics [1, 36] nor concrete best practices for implementation [37]. Indeed, the US NIH FIC Bioethics Program does not endorse any particular instructional program but encourages experimentation with different models [1]. Thus, bioethics training programs are diverse, as are the courses covered and the duration. Despite the increase in the number of training programs in LMIC and the demonstrated success of some [6, 24], there is a paucity of curriculum content and detailed methods for implementation in resource-limited contexts. Two decades have passed since the first NIH FIC research ethics training awards for African scholars and professionals, but there is still a dire need for more programs that are tailored to unique local needs [38]; the employability of bioethics graduates is a challenge in some countries [11]; and individuals with the capacity to teach research ethics are lacking. Two primary questions emerge: 1) What models are available for long-term training to address emerging needs of research ethics capacity for biomedical and public health research in disease-burdened LMICs? 2) How do we balance the necessary training for dual specialization in public health and research ethics, necessary for graduates to be competitive on the job market without crowding the curriculum? The Mbarara University Research Ethics Education Program (MUREEP) at Mbarara University of Science and Technology (MUST) in southwestern Uganda sought to respond to these challenges. In 2018, we developed a research ethics track and combined it with an existing 2-year public health master’s degree program (MPH-RE) in the Department of Community Health, Faculty of Medicine at MUST.. The purpose of this paper is to describe this MPH-RE program and our five-year experience of implementation. We hope that the details of the model shared in this paper will be useful to training programs that have related strategies or those that may consider adapting it to their contexts, and will also stimulate discussion on enriching such strategies in similar settings.

## Methods

A PubMed search was made using “research ethics training”, bioethics, and Africa, generating 117 articles. From these, we searched for literature on long-term training of African scholars to develop research ethics capacity, the priority areas, and best practices of bioethics or research ethics education programs. Long-term training was defined as postgraduate training in bioethics or research ethics education program with a duration of at least 3 months culminating in the award of a certificate, diploma, or degree [39]. Our analysis was from 2000 to 2017, spanning the time before the MUREEP, to identify existing training programs meeting our local needs, namely to develop research ethics capacity in a region with a high disease burden that is attracting biomedical and public health research. The Strengthening Bioethics Capacity and Justice in Health program, a collaboration between the University of North Carolina and the University of Kinshasa in the Democratic Republic of Congo, implemented a MPH curriculum with a concentration in research ethics for francophone countries in Africa [6], but we did not find detailed literature about the program. Of particular interest were the first five institutions that received the NIH FIC’s grants for research ethics education and curriculum development programs for LMICs in 2001 as they shaped bioethics education in Africa. For the five institutions, we used two other sources of data in addition to what is published: the NIH RePORTER and program websites. Relevant missing information about the program at CWRU and the SARETI was obtained via email to the official contacts.

Data about MUREEP are for 5 years (April 2018 to January 2023). We used curriculum materials, trainees’ post-training evaluations, applications to the program, annual reports, and the external evaluation for this paper. The MPH-RE curriculum described in this paper was accredited by the National Council for Higher Education in Uganda. We use the term “curriculum” broadly in view of the purpose of this paper to refer to the academic courses and activities of a program in an institution and the key inputs to guide teaching for students to gain proficiency in the expected knowledge and applied learning skills. Additional information was gathered from the MUREEP records and annual reports.

### Description of the Mbarara University research ethics education program (MUREEP)

With funding from the National Human Genome Research Institute and the Fogarty International Center (FIC) of the US National Institutes of Health in 2018, MUST in southwestern Uganda, collaborating with Case Western Reserve University (CWRU), Cleveland, Ohio, in the USA rolled out the MUREEP. The main aim of the MUREEP is to build multi-level research ethics capacity in Uganda, specifically by: 1) developing and implementing a curriculum for research ethics education and training, leading to (a) a Master of Public Health degree with a focus on research ethics and (b) a repertoire of short courses to build research ethics capacity of Ugandan investigators, REC members and administrators; 2) create a critical mass of teachers and mentors for sustainable research ethics training in southwestern Uganda; and 3) strengthen the expertise and functioning of Research Ethics Committees (RECs) at MUST and other local institutions. Implementation of aim 1 (a) would produce 12 students, and a minimum of 525 short course trainees for aims 2 and 3. The collaboration covered strategic planning, program leadership, with MUST being the primary awardee, curriculum development and implementation, faculty, and mentoring.

#### MUREEP MPH-research ethics curriculum

We are implementing a well-structured curriculum designed to lead to the award of a Master’s degree in Public Health with a concentration in Research Ethics (MPH RE). Specifically, MUREEP added a new track, Research Ethics, to an existing Master of Public Health program offered in the Department of Community Health, Faculty of Medicine at MUST.

##### The MPH research ethics program structure

Our two-year MPH-RE curriculum is broadly structured into five major components: 1) MPH core courses; 2) MPH-Research Ethics courses; 3) non-degree short courses; 4) practicum and externships, and 5) research and dissertation.

##### MPH core courses

The curriculum has 9 core MPH courses of 60 contact hours each and fundamentals of clinical trials of 45 contact hours (Table 1) to develop the competences of public health bioethicists for a high-disease burdened country (Table 1). The goal is, first, to equip students with knowledge, skills, and attitudes to be sensitive and responsive to health inequalities among populations, and secondly, to bring to light the reasons underlying research, especially in rural communities.

**Table 1:** MPH-Research Ethics Courses and Credits^a^

MPH-RE Course Structure				
Year 1			Year 2	
Semester 1	Semester 2	Recess	Semester 1	Semester 2
Coursework	Coursework	Coursework	Coursework	
Epidemiology (4) Biostatistics (4) Research Methods & Survey Design (4) Health Promotion and Community Involvement in Health (4)	Health Policy Planning and Management (4) Infectious Disease Control (4) Maternal, Child Health and Nutrition (4) Environmental and occupational Health (4) Foundation of Research Ethics (3)	Research Proposal Development and Presentation (5) Responsible Conduct of Research (3) Regulation of Research and Policy (3) Critical reasoning in Research Ethics (3)	Program Monitoring, Evaluation and Quality Improvement (4) Fundamentals of Clinical Trials (3) Public Health Research Ethics (3) Practicum and Externships (3) - REC and UNCST placements - Community engagement Skills for Teaching Research Ethics (3)	Grant writing (3) Research Independent mentored research project in research ethics - Research seminar (5) Dissertation Development and Defense (5)

^a^Credit units in parentheses, 1 credit = 15 contact hours (1 hour lecture = 1 contact hour, 2 hours practical/tutorial = 1 contact hour); *REC* Research Ethics Committee, *UNCST* Uganda National Council for Science and Technology

To equip students with skills to design epidemiological studies, we begin by introducing the fundamental concepts of epidemiology and the scientific methods of investigation and problem-solving that are used to get to the root problem of disease in populations. Students learn how to critically appraise study designs (research methods; 60 contact hours); how to use statistical software to analyze the generated data, interpret it, and make practical recommendations. In biostatistics (60 contact hours), emphasis is put on various forms of data, scales of measurement, and data conversion between different scales. Research methods and biostatistics courses, which are covered in semester 1, provide a strong foundation for the development of research proposals. Students have a ‘skills application’ one-week residential placement in the community for health promotion and community involvement.

The second year of study is a practical immersion experience, working with RECs, practicing teaching research ethics, developing skills for grantsmanship, and conducting mentored research in research ethics.

##### Mode of delivery

In keeping with the inter-professional education approach in the Faculty of Medicine at MUST, MPH-RE students study epidemiology, biostatistics, research methods and survey design with other health profession students (Master of Medicine, Master of Nursing in Critical Nursing, Master of Pharmacy in Clinical Pharmacy Practice, and Master of Medical Laboratory Sciences). This combined class provides a unique setting for learning. Given the diversity of education backgrounds for ‘traditional MPH’ and MPH-RE students, sufficient time is dedicated to lectures, self-reading, journal clubs, small group discussions, and presentations.

##### MPH research ethics courses

The courses carry three credits (45 contact hours) each. Course material is adapted for local context and culture, and for -resource-limited settings. We begin with the philosophical foundations of research ethics. Given the increasing genetic/genomic and infectious disease research on the African continent, ethical issues are extensively covered under public health research ethics, regulation of genomic research, and controversial ethical issues in biomedical and biotechnology research. We use case scenarios from ongoing local and published investigations in addition to didactic lectures, pre-recorded videos, and group discussions.

##### Foundations of research ethics

The course introduces students to philosophical theories, fundamental ethical principles and theoretical approaches to bioethics, and their application in clinical, biomedical, and public health research. Relevant international guidelines pertaining to ethical issues and legal aspects in the context of Uganda are covered. To have a connection between theories and practice, we use readings and cases to explore professionalism, ethical challenges associated with review of research protocols by RECs, informed consent, notions of risks and benefits, the distribution of health resources nationally and internationally, and justice in global health research. Trainees in the health science professions discuss scenarios for group discussions, to learn how to balance physician expertise and respect for patient autonomy and ethical clinical decision-making among vulnerable populations. Cultural underpinnings of ethical principles are addressed with special attention to both western and non-western approaches to conceptualizations of ethical theory. As noted by others, applying bioethics in sub-Saharan Africa still remains challenging given the diverse cultural values, beliefs, indigenous faiths, different religions with those of the Christian faith residing in the same settings as Muslims, and the conflicting perception of the international principle of identity and authenticity [2] and healthcare practices. Our method is to contextualize the case studies to settings and cultural values and incorporate the essential two dimensions of bioethics (western and non-western) so that ethical values are not viewed as paternalistic but also avoid the extremes of community and solidarity ethics, the *ubuntu ethics* [40].

##### Public health research ethics

The course (45 contact hours) builds on the concepts covered in the MPH core courses of first semester. This course puts emphasis on ethical issues in infectious disease research, which is our biggest challenge; genomics and genetic studies among populations in resource-limited settings who often have a limited understanding of the concept of research; and vaccine research from the community point of view. Ethical challenges in maternal and child health research, and research ethics with sexual minorities are covered. One of the senior MPH-RE student’s research focused on understanding the community’s and research participants’ perceptions and knowledge of vulnerability in research – in the Ugandan context. Ethical challenges in community engagement for public health research, and research conducted during outbreaks, which are recurring in Uganda [29–32] are covered while emphasizing a social justice approach.

##### Critical reasoning in research ethics

For the MPH-RE graduate, the ability to think clearly and reason well about ethical issues is a fundamental aspect of training to be able to deal with ethical dilemmas and arrive at morally acceptable answers and decisions. This course examines three important aspects of critical thinking: namely, the ability to understand and evaluate arguments; the ability to make well-reasoned decisions; and the ability to be open-minded. The goal is to develop students’ skills for critical reasoning applied to theoretical and empirical literature addressing ethical concerns in scientific research, with particular interest in genomics and biotechnology.

#### Non-degree short courses

The MUREEP offers four short courses for faculty, graduate students, research staff, REC members, and administrators at MUST and other institutions, mostly in southwestern Uganda. The short courses (regulation of research and policy, responsible conduct of research, skills for teaching research ethics, and grant writing) are mandatory for MPH-RE students, each covering at least 30 hours. A previous evaluation of the curricula of 13 Fogarty research ethics training programs showed that similar topics are covered, although in some cases not as courses, while for some, like grant writing and teaching skills, the duration is not specified [37]. At the time of writing this paper, 609 trainees had completed various short-term research ethics courses offered by the MUREEP (Table 2). The majority of trainees (382, 62.7%) attended the Responsible Conduct of Research (RCR) course, followed by regulation of research and policy (68, 11.2%).

**Table 2:** Participants of the MUREEP non-degree research ethics courses

Course	Trainees	No. of trainees
Critical Reasoning in Research Ethics	MPH-RE students	17
Foundations of research ethics	MPH-RE students, faculty, research assistants, REC Chairperson	44
Grant writing	MPH-RE students, faculty, non-MPH-RE postgraduate students, Residents, researchers at MUST and BSU	55
Public Health Research Ethics	MPH-RE students	17
Regulation of Research and Policies	MPH-RE students, REC members and administrators, regulatory officer, administrators, researchers, doctoral students, faculty at MUST, BSU, KAB and KIU-WC	68
Responsible Conduct of Research	MPH-RE students, non-MPH-RE postgraduate students, Residents, faculty, research assistants, REC members and administrators, data management officers, researchers, doctoral students, staff at MUST, BSU, KAB and KIU-WC	382
Skills for teaching Research Ethics	MPH-RE students and selected faculty from BSU & KAB	26

Key: MPH-RE – Master of Public Health with Research Ethics; REC – Research Ethics Committee; BSU – Bishop Stuart University, KAB – Kabale University, KIU-WC – Kampala International University-Western Campus

##### Regulation of research and policy

This is primarily important for the promotion of ethical research and the protection of research participants, and it details the roles and responsibilities of various committees and bodies involved in research oversight and regulation. MPH-RE students critique international and national guidelines for ethical conduct in research with human subjects to identify gaps that need policy development or revision. The course is also open to REC members, administrators, graduate students, and other interested persons. Trainees role-play ethics committee deliberations and discuss case scenarios.

##### Skills for teaching research ethics

This course targets those involved in teaching research ethics or bioethics at MUST and other local institutions of higher education as science faculty transition from an *ethics compliance mindset* to promoting an ethics learning to enhance and advance science [41]. The course is mandatory to MPH-RE trainees. Participants are introduced to methods and resources for teaching bioethics as well as teaching trends, challenges, and opportunities in that field [42]. Trainees are first introduced to the theories that influence teaching and learning [43] before tackling the learning environment, planning and teaching sessions, teaching skills, forms of teaching, and assessment. As a practicum experience, MPH-RE trainees participate in teaching research ethics at MUST and other institutions and make presentations in seminars and research ethics conferences.

##### Responsible conduct of research

The course is open to graduate students, investigators, faculty, REC members, staff, and anyone involved in research at MUST and other institutions in south western Uganda. It builds on other courses in research ethics with additional modules on bioethics in the Ugandan and sub-Saharan African (SSA) contexts. The course covers 16 broad areas including the mandatory topics provided in the NIH guidelines on instruction in RCR [44]. Didactic lectures are complemented with case study discussions, education videos, and field visits to research institutions and laboratories, clinical research sites, the MUST Animal Research Facility, and the MUST REC Office.

##### Grant writing

This 5-day course provides an overview of the core elements of grant writing as part of the overall research enterprise. The expected learning outcome is the preparation of a standard and competitive grant proposal for submission to a funder. It is an inclusive inter-professional education activity that promotes student-initiated collaborations among themselves and with faculty.

#### Practicum and externship

The MPH-RE students take part in three types of practical and externships to apply the knowledge obtained from the foundational bioethics and research ethics courses to develop skills needed to serve on RECs. First, students join the MUST REC to observe meetings and participate in mentored reviews of research protocols. Human research participant issues in resource-limited settings are unique in many ways including working with illiterate, poor populations which increase their vulnerability. We believe that the “public health research ethics” and “clinical trials” courses of the program will enable students develop capacity to review protocols of international research, and genomic and genetic studies because of issues such as complexity of genetic concepts and implications of study findings for family members and communities [45]. Second, students undertake externships either with a high-load REC or at national regulatory offices. Third, students participate in study site monitoring visits. Fourth, the students have online observership of CWRU Institution Research Board deliberations. The approach is to build their competence to lead and serve on RECs [34].

#### Research project and dissertation

Research proposal development begins in the recess of the first year, followed by a mentor-guided research project in research ethics in the second year. Students must write a dissertation for examination and publish or present their findings at a conference relevant to the field of research ethics. The Program provides the students with a research stipend and ethics review fees, in addition to a laptop to support their journey in becoming responsible research ethics experts.

#### Research seminar

One important component of a research project is the successful defense of the dissertation. This is mandatory for all postgraduate students and is assessed. The research seminar for the MPH RE students aims at imparting methods and techniques for how information obtained from literature review, research project and dissertation is condensed and written in PowerPoint and manuscript.

#### MPH-RE students

Trainees who are awarded the MUREEP fellowship are competitively selected to ensure that the knowledge and acquired skills are quickly put to use for the early success of the program. We selected applicants using pre-determined scoring criteria. Between October 2018 and November 2022, 20 trainees (14 full scholarships, 04 partial scholarships, and 02 self-sponsored) with diverse professional backgrounds were admitted to the MUST MPH-RE program (Table 3). The targeted number of enrolled students was 12 over the five-year period of funding. As of the writing of this paper, MUREEP has graduated 3 Masters students, while 4 submitted dissertations for examination; the rest are at different stages of their mentored research projects (Table 3). One alumnus and two MPH-RE students are serving on institutional RECs; six are faculty members in four institutions; others are in clinical and public health research and community work. While the MUREEP MPH-RE program does not have any drop-outs, the completion rate has been low in the first 5 years of program implementation. However, existing literature shows that evaluations of bioethics training programs have been, on average, after 12 years [1, 6, 37], so it may be premature to draw conclusions on the training outcomes of MUREEP in the first 5 years. The pioneer MPH-RE class and one student of cohort 2 graduated in 2021, following the reopening of institutions, and were met with high demand on the job market. They either received new appointments or were promoted and assigned higher-level tasks at their places of work (on REC, university faculty, leadership positions on a clinical trial, and in a research collaborative in Uganda). All were supported to present their research findings at international conferences, and their manuscripts are undergoing peer review in international journals. With disruptions of training and suspension of research activities 14 months after the commencement of the program to contain the coronavirus disease 2019 (COVID-19) pandemic, it is difficult to determine what more would have been achieved by individual students or by the program.

**Table 3:** Characteristics of MPH-RE students 2018–2022

Cohort	Academic year	No. of students	Education background	Outcome/current status
1	2018/2019	2	Education Psychology	Graduated
			Nursing	Graduated
2	2019/2020	6	Human Medicine	Submitted dissertation for examination
			Nursing	Graduated
			Nursing	Submitted dissertation for examination
			Gender and applied women health	Collecting data
			Development studies (monitoring and evaluation)	Waiting REC approval
			Community Health	Collecting data
3	2020/2021	5	Environmental Science	Completing data collection
			Physiotherapy	Submitted dissertation for examination
			Nursing	Collecting data
			Development studies	Submitted dissertation for examination
			Development studies	Collecting data
4	2021/2022	4	Medicine	Waiting REC approval
			Business Administration	Waiting REC approval
			Nursing	Proposal development
			Science Education	Proposal development
5	2022/2023	3	Development Studies	Proposal development
			Physiology	Proposal development
			Education	Proposal development

#### Faculty

The interdisciplinary nature of the public health and research ethics curriculum demands faculty from different disciplines. In addition to the existing human resources for the MPH program, the MUREEP faculty in Uganda and our collaborators at CWRU have specializations in the fields of bioethics, medicine, social sciences, law, similar to other programs in HIC and LMIC [37] in addition to biochemistry, epidemiology, pharmacology, pharmacy, biotechnology, genetics and genomics, anthropology, divinity, psychology, and education.

#### Other training activities

##### Journal club

This is the single most important academic activity that provides regular interaction between students, alumni, and faculty. Every 2 weeks during semester time, students have a hybrid journal club lasting 60 to 90 minutes. Three-month schedules are drawn for the journal club to allow students to look for relevant articles, either in line with their research project or a topic of interest. The journal clubs are intended to sharpen students’ critiquing skills, appraise research in research ethics, and improve presentation and communication skills.

##### Mentorship and career development workshop

While this is not part of the research ethics education curriculum at MUST, MUREEP conducts a five-day mentorship and career development workshop to improve the skills of MUST faculty to mentor colleagues and students while achieving professional success. There is demand for the workshop, but attendance is capped. Forty-three individuals have attended the two workshops that have been conducted.

## Discussion

Research ethics education keeps evolving to adapt itself to contemporary ethical dilemmas. With the new challenges in public health like the double burden of malnutrition [33], the occasional outbreaks of health emergencies and emerging and re-emerging vector-borne zoonotic diseases [46, 47], which necessitate having specific skills for One Health approaches, the complex multi-country clinical trials [34], and some unique ethical issues with sexual minorities in Uganda with implications for public health and bioethics [48], the MUREEP was started to build multi-level research ethics capacity at MUST and in three neighboring institutions (Bishop Stuart University, BSU in Mbarara district; Kampala International University-Western Campus, KIU-WC, Ishaka in Bushenyi district; and Kabale University, KAB in Kabale district, southwestern Uganda).

With the new trends in biotechnology and emerging health technologies such as artificial intelligence and data science [49, 50], there is a need to develop competence for quality scientific and ethics review of complex protocols in Uganda [34], and for every high-volume REC in LMIC [35]. Bioethics education programs in SSA should broaden the scope of eligibility for higher-degree training in research ethics to keep up with the global demands of bioethics graduates.

The MPH-RE program is different from “bioethics”, which is a distinct academic and professional discipline in Western universities [2], like at CWRU, JHU, and the University of Toronto. Bioethics provides strong philosophical foundations for navigating different ethical dilemmas in different fields, such as clinical ethics, environmental ethics, population ethics, and research ethics. Our program focuses on the “research ethics” component of bioethics, given the high volume of research being conducted in SSA by investigators from HIC and the ethical challenges that come with it. However, the philosophical theories ethics, fundamental ethical principles and theoretical approaches to bioethics, and their application in clinical and public health research covered in the Foundations of Ethics course prepare students apply knowledge to healthcare practice.

Similar to successful research ethics training programs out of Africa, such as the International Research Ethics Training Program (IRETP) at CWRU (2000–2016) and the University of Toronto Masters of Health Sciences in Bioethics, International Stream, UTMBIS (2000–2012), the MUREEP offers training in international research ethics to professionals from a variety of disciplines, all with a course on pedagogy and mentored research in research ethics. However, the three programs differed in the main focus of the program, duration, and cumulative number of trained individuals to address the need for research ethics capacity in LMIC. Both the IRETP and the UTMBIS focused on bioethics, whereas the MUREEP focuses on public health and research ethics. Similar to the UTMBIS, the MUREEP MPH-RE program is for 24 months, while the Master of Bioethics CWRU program was for 1 year. Both IRETP and the UTMBIS received trainees from at least four different countries, contributing immensely to the establishment of research ethics frameworks in their home institutions. The MUREEP offers multi-level research ethics training through its Master’s program and non-degree short courses, fostering local capacity development for students, REC members, investigators, and staff.

The MUREEP MPH-RE model is similar to what the SARETI at the Universities of Pretoria and KwaZulu Natal offered from 2001 to 2013. In addition to implementing a 1-year MPH program, SARETI has had experience in diversifying the teaching of research ethics in other graduate programs, including doctoral level, possibly to address the evolving training needs in Africa. The discontinuation of SARETI’s MPH program in 2013, followed by the remarkable enrolment and completion of training for trainees on the MSc. Social Sciences (14 for MPH; 36 for MSc. SocSc over 17 years) suggest that programs should have awareness of evolving training needs, and respond by designing and implementing appropriate curricula. Unfortunately, we did not find in the existing literature detailed reviews comparing SARETI’s MPH and MSc. SocSc. programs to identify areas for improvement of MPH RE programs. Nevertheless, having a rich social perspective is likely to help graduates understand the more complex cultural and social factors that still pose a challenge to teaching bioethics in Africa. The SARETI program offered two modules on culture and morality [6]; the MUREEP MPH-RE curriculum does not cover them as distinct courses, but indigenous and cultural issues in research are covered in case studies, in the critical analysis of bioethical issues (critical reasoning in research ethics course), and during the residential community placement to prepare them as future members of RECs and regulatory bodies. We address ethical issues, such as who is authorized to provide consent and those associated with giving blood or other biological samples. We are, however, careful not to overload the research ethics curriculum.

As long as there is ongoing student interest, adequate promotion of the MPH-RE track, and ongoing support from the department of community health leadership, the integration of research ethics education into the current MPH program through the creation of an MPH-RE track is a formula for sustainability. We believe that the MUREEP 2-year MPH-RE model can be adapted and used elsewhere in developing countries to increase the relevance of research ethics education for sustainable development. Importantly, our research ethics education curriculum is addressing the challenge of career path and employability that has been reported by some training programs offering a Master of Arts in bioethics or a Master of Science in health research ethics alone [11]. Like in many other research ethics or bioethics education programs in and outside Africa, the MUREEP MPH-RE students are taught and empowered to become leaders in research ethics; equipped to teach research ethics, and conduct empirical research [37]. Since 2021, the MPH-RE students (particularly cohorts 1 and 2) have been involved as trainers alongside MUREEP faculty in a number of RCR trainings in different venues and with different audiences, a model worth reproducing. The alumni are currently serving as instructors of some research ethics courses in the program, a capacity-building approach for academia. While MUREEP has so far graduated a few students, with some awaiting the completion of their dissertation examinations, the new job appointments and/or promotions of the alumni are evidence of the employability of our graduates. The graduates are qualified to serve as leaders and members of RECs, as public health experts and perform duties such as research coordination, advocacy, project management, community engagement, and monitoring and evaluation. We think that the MPH-RE approach has the potential to serve similar contexts in Africa with a high disease burden and complex clinical trials to promote responsible conduct of research while supporting the workforce in other areas of health research and public health emergencies [51]. At an appropriate time, it will be necessary to evaluate this strategy at MUST in Uganda together with similar programs like the Johns Hopkins University-Addis Ababa University Research Ethics Training Program in Ethiopia and the United States-Mali Research Ethics Training Program in Mali, which started in 2020.

Interdisciplinary approaches are being promoted in academia, and many research teams consist of experts from different disciplines, ranging from applied sciences and technology to life sciences, social sciences, and the humanities. Integrating research ethics education in a range of health, behavioral, and natural sciences should be considered [42] to prepare scholars for multidisciplinary research with its demands for integrity, compliance with regulations, rigor [52], and to serve on high-volume RECs reviewing complex studies. We think that it is attainable, particularly if research ethics is added to a curriculum as an optional track. It leverages existing infrastructure, provides on-going opportunities to examine ethical issues contextualized in commonly known disciplines for future professional roles, and helps students exploit the synergies of the disciplinary content of the curricula.

Creating and maintaining a culture of mutual trust and commitment to responsible research in institutions requires that all stakeholders at different levels (Table 2) receive some form of training [53]. DuBois and Antes discussed five dimensions of research ethics: “(1) normative ethics, which includes meta-ethical questions; (2) compliance with regulations, statutes, and institutional policies; (3) the rigor and reproducibility of science; (4) social value; and (5) workplace relationships”, each with unique stakeholders that need research ethics training to create a climate of research integrity in an institution [54]. This climate is a culture of responsible conduct of research and compliance with acceptable research practices in an institution [55]. MUREEP has established itself as a premier program offering RCR training in southwestern Uganda (Table 2). The impact of this is yet to be established.

Our non-degree short courses are open to all postgraduate students, REC members, researchers, faculty, administrators, and regulators. Despite the disruptions of the COVID-19 pandemic that affected the delivery of courses, the large number of trained individuals (609) and an additional 43 participants who attended the mentoring and career development workshops are way above what we originally anticipated (525 trainees). We do not provide the figures of trainees as heuristic implication of the effectiveness of the program but, rather as an initial indicator that there is increased awareness and a huge need to develop research ethics capacity in institutions in southwestern Uganda.

Having a good curriculum is good, but ensuring that there is sustained cognitive and affective learning is another thing and challenging. We observed that, in addition to practical externships with RECs and other regulators, and in the community, affective student learning is enhanced by well-coordinated field visits to exemplary researchers and research organizations, particularly those who have succeeded in collaborative work and research involving vulnerable populations. Post-training evaluations by our trainees show that field trips increased their interest in research ethics and provided meaningful engagement with individuals who encounter ethical issues in authentic environments. We believe that it is an important approach for developing scientific virtues [56], as trainees can recall those experiences and their novel discoveries and observations long after the visits.

While our original method of delivering short courses in five-day blocks, which was used elsewhere [7], was justified at the beginning of the program because of the need for foreign expertise, it proved to be physically straining for both students and the local faculty. In one of the evaluations of the program conducted in 2022, students reported that the volume of information in the courses during the 5 days was often burdensome, making retention and comprehension difficult. This revealed a weakness in the structure of the MPH-RE curriculum, which was addressed with a spread-out of courses over several months except RCR attended by diverse groups of people from different institutions in the region. We also observed that certain assessments can be compromised due to time constraints [57] or are assessed discretely rather than as integrated learning. However, some programs in developed countries have reported successful block teaching models [58]. For training programs that plan to integrate research ethics courses into other high-degree curricula, we suggest careful planning of the delivery of the courses with minimal deviation from the system of teaching used in the institution.

Our spiral MPH-RE curriculum recognizes the importance of in-depth training in research methods and biostatistics. The importance of a biostatistician on RECs has been discussed before [59], and bioethics education programs should intentionally prepare their graduates for this task. We are using a spiral training approach to deepen and reinforce what has been taught in previous encounters and to ensure knowledge and skill retention in these two important courses. The concepts and applications of research methods and data analysis are integrated into several courses. Students are expected to appraise research methods in RCR, critical reasoning, when reviewing protocols during REC externships, in research seminars and grant writing. They apply statistical methods to public health, behavioral, and clinical research, and interpret statistical output resulting from data analyses.

Bioethics students should acquire advocacy skills, appreciate the importance of working with multiple stakeholders, and have the ability to identify a policy solution. A research ethics graduate should have skills for ethical engagement over controversial issues and social justice. Some authors have argued that public health advocacy is necessary to foster research integrity by ensuring the disclosure of important results and their dissemination by all parties, including national governments [60]. Indeed, health system advocacy and science advocacy are thought to play a synergistic role in informing policy on systems that impact clinical and community-based research [61]. An early evaluation of 13 Fogarty-funded bioethics programs that offered at least 1 year of training reported that 2/13 were not offering instruction in health policy, while 4/13 covered it in less than 1 hour [37]. We recommend that that instruction in health policy and advocacy should be included in research ethics programs because advocacy skills are necessary for developing strong health research governance structures in Africa [62] and may be important drivers of sustainable development goals.

We think it prudent for students to be introduced to disease burdens and risk in populations and communities, and how it fuels research before they consider ethical issues in public health research in Year 2.

We use a flipped classroom approach for the delivery of the critical reasoning course for students to realize that managing ethical dilemmas goes beyond knowledge, to analytical reflection, reasoning [63], and critiquing one another’s opinions. Students are able to ask more questions in class and to practice interpersonal skills as they consider different points of view of complex situations.

## Conclusions

It is not the intention of this paper to provide the impact of the MUREEP or the MPH Research Ethics degree program but rather to share a model that can be reproduced or adapted to develop bioethicists with specific applied learning skills in countries with a high burden of disease, like in much of SSA. Given that there is no “gold standard” for teaching bioethics, it is important to define best practices, which necessitate sharing methods of implementation in similar contexts. The comprehensive MPH component of our program seems to be an added advantage that opens up more job opportunities for the graduates and a breadth of fields for specialization with a component of research. We believe that this exemplar for teaching research ethics can be modified for other specialties, to meet specific institutional or national needs, or even to implement innovative teaching philosophies and approaches.

### Lessons learned

There should be continuous and systematic internal evaluation of the program to inform MUREEP’s direction over time. In the first 5 years, we used various mechanisms such as short courses, workshops, online classes and in-person training. Our experience shows that in-person courses delivered gradually over a few months is the best option as it provides students more time to digest novel and large volumes of information. Programs that have or plan to develop curricula with research ethics as a track in another academic program ought to “integrate” it into the teaching early in the first semester and perhaps add relevant research methods, especially for qualitative studies, in the second semester. In our case, it means teaching some core MPH courses and MPH-RE courses at the same time for students to make immediate connections between public health practice/research and research ethics. This requires careful balancing of the semester credit load so as not to overload the curriculum. While infrastructure may be a challenge for many institutions in resource-limited settings like ours, programs must be developed with adults in mind to achieve their goals and be effective. Adult learning requires space, and so do highly participative activities like case studies and small group discussions. The venue for training matters a lot, especially for professionals and university administrators.

### Challenges

The first challenge was the initial scarcity of educational resources, which did not come from Europe or the United States and were relevant to the Ugandan context. This was mitigated by the use of Africa-focused case studies. Trainees are encouraged to share their working experiences. We also use cases from researchers and clinicians working in Uganda. Students also develop case studies as practicum in the skills for teaching course. Promoting bioethics in institutions where the program is not well understood is difficult and requires supportive institution leadership and the department hosting the program. There may be a disconnect between knowledge about research provided by the MPH component of the MPH-RE program and the research that MPH-RE students are expected to perform for their dissertation in the department and among students in their first year. As already mentioned, it may be because of the delay to introduce the research ethics courses. However, the biweekly journal club that was started in 2020 is helping students identify research gaps, critique published work in the field of research ethics, learn how to respond to questions, and test the limits of their knowledge. As elsewhere, COVID-19 pandemic disruptions affected the timely delivery of courses, the mobility of foreign faculty to MUST, and caused unprecedented delays in the ethics and scientific review process of students’ research proposals. However, it provided the opportunity to develop online teaching materials and pre-recorded lecture videos, increasing flexibility in teaching and learning. Later, these platforms became students’ helplines to support learning and mentoring–a challenge turned into opportunity and resilience. We recommend that continuing and new bioethics training programs in Africa should develop and/or strengthen their contingency plans based on scenarios of emergency situations and catastrophes to minimize disruptions in teaching and learning.

Formalizing mentoring with specific pre-outlined goals for career development is still a new concept in many institutions in sub-Saharan Africa and is commonly attached to internationally funded programs. Programs with a small pool of competent local mentors need to support senior students and recent bioethics graduates to serve as peer mentors.

### Continuous improvement and future directions

We plan to review and revise the curriculum in 2024 for the long-term direction of the program in fulfillment of the requirement by the National Council for Higher Education, the regulating body in Uganda. We shall work towards the complete integration of research ethics into the core MPH courses early in the program. As the only training program for research ethics in southwestern Uganda, we have a responsibility to prepare the next generation of professionals ready to tackle the anticipated challenges related to new technologies. We plan to broaden the multidisciplinary nature of the program. Previously, some Fogarty-funded programs were in favor of candidates from the health and social sciences [37]. Our focus is to extend and consolidate the delivery of research ethics education in Uganda, work towards integrating mentoring into MUST, establish a research ethics advisory center, and develop a PhD program.

## Data Availability

All data generated in this study are included in this published article.

## Abbreviations

AIDS: Acquired immunodeficiency syndrome
ARESA: Advancing Research Ethics Training in Southern Africa
BSU: Bishop Stuart University
COHRED: Council on Health Research for Development
CWRU: Case Western Reserve University
FABTP: Fogarty African Bioethics Training Program
FIC: Fogarty International Center
HIC: High income countries
HIV: Human immunodeficiency virus
IRENSA: The International Research Ethics Networks for Southern Africa
IRETP: International Research Ethics Training ProgramJHU John Hopkins University
KAB: Kabale University
KIU-WC: Kampala International University-Western Campus
LMIC: Low-and-Middle income countries
MHSc: Master of Health Sciences
MPH: Master of Public Health
MPhil: Master of Philosophy
MSocSc: Master of Social Sciences
MUREEP: Mbarara University Research Ethics Education Program
MUST: Mbarara University of Science and Technology
NIH: National Institutes of Health
RCR: Responsible Conduct of Research
RE: Research Ethics
REC: Research Ethics Committee
SARETI: South African Ethics Training Initiative
SSA: Sub-Saharan Africa
UTMBIS: University of Toronto Masters of Health Sciences in Bioethics, International Stream
